# With a Little Help from my Enteric Microbial Friends

**DOI:** 10.3389/fmed.2015.00030

**Published:** 2015-05-11

**Authors:** Ben Berkhout

**Affiliations:** ^1^Laboratory of Experimental Virology, Department of Medical Microbiology, Center for Infection and Immunity Amsterdam (CINIMA), Academic Medical Center, University of Amsterdam, Amsterdam, Netherlands

**Keywords:** norovirus, mouse mammary tumour virus, LPS, poliovirus, reovirus, microbiology

## Abstract

Although the disciplines of bacteriology and virology frequently come together in the setting of a diagnostic medical microbiology laboratory, the two scientific fields are usually miles apart. The microbiologists basically form two non-overlapping groups of scientists, the bacteriologists and virologists, which go to separate meetings and do not easily intermingle. Some recent research findings about elegant virus–bacterium interactions may change this situation. Obviously, interactions between these two microbes can occur only when they colocalize, which most likely occurs in the gut/intestines where 10^14^ commensal bacteria reside (the microbiota). We review findings on the following enteric microbial tandems: norovirus *– Enterobacter cloacae*, mouse mammary tumor virus (MMTV) – bacterial lipopolysaccharide (LPS), poliovirus and reovirus – intestinal bacteria. The close bacterium–virus interplay may also present options to develop unique therapeutic strategies for those infected, and to prevent further virus spread, and thus minimize the risk for the community.

## Norovirus Needs a Bacterium to Enter Cells

Members of the norovirus and sapovirus genera of the *Caliciviridae* family of small positive stranded RNA viruses are infamous for causing outbreaks of gastroenteritis (stomach ache, nausea, and diarrhea) in nursing homes and on cruise ships. Noroviruses have been estimated to be responsible for 85% of these outbreaks, and there is no cure or vaccine available (1). Although the first norovirus was identified nearly 50 years ago, it remained impossible to replicate human noroviruses in cell culture and in small animal models in the laboratory. As a result, progress on the study of human norovirus pathogenesis and the molecular replication mechanisms has lagged behind that of other positive stranded RNA viruses, and development of antiviral drugs has also been hampered. The absence of appropriate test systems also meant that the cellular tropism for this enteric virus remained unknown. The best surrogate system is the murine norovirus that does replicate in cultured cells and mice. Several early findings suggested that murine norovirus strains require B cells (2–4). In particular, mice lacking B cells exhibit reduced viral titers and the murine virus can be detected in intestinal B cell zones in wild-type mice, but how these findings relate to human noroviruses remained unclear.

Jones et al. recently reported that human norovirus needs help from *Enterobacter cloacae*, environmental bacteria that may reside in the gut of hospitalized patients who received antibacterials, to replicate in cultured human B cells (5). The key finding was that unfiltered stool samples can initiate norovirus replication, whereas filtered samples demonstrate a firm infection block. This suggested that a critical factor is removed during the standard practice of filtering human stool samples over 0.2 μm membranes to remove bacteria, hinting at help from the bacterial side. *E. cloacae* became the prime suspect because it was known to bind norovirus (6). This interaction involves glycan structures on the bacterial surface that mimic blood type antigens on red blood cells, and synthetic glycans can reproduce this effect. The virus–glycan interaction was proposed to facilitate norovirus attachment to cells, as individuals that lack these antigens are resistant to norovirus infection (7, 8). To demonstrate bacterial involvement, filtered stool samples were mixed with *E. cloacae*, which reestablished their ability to infect human B cells (5). The effect is specific for *E. cloacae* as neither *Escherichia coli* nor the bacterial product lipopolysaccharide (LPS) rescued norovirus infectivity.

Complementary results were obtained for the mouse norovirus (5). This virus also infects B cells *in vitro*, and treatment of norovirus-infected mice with antibiotics reduced the viral load. A new twist to this story was added very recently in two studies, suggesting that the gut microbiota may interfere with antiviral innate immunity by quenching interferon-lambda (IFN-λ) signaling by an as-yet undiscovered mechanism (9, 10).

It is clear that this new culture system for human norovirus removes a major hurdle for many studies that were previously impossible. To reduce the disease burden, better prevention and control strategies for noroviruses are badly needed, and their development is greatly facilitated by a simple culture system. The recent findings also raise new research questions. First of all, mechanistic details are still lacking on how glycans on the bacterial surface, but also free synthetic glycans, stimulate norovirus attachment to B cells. Second, there may be significant variation on the microbial side. Differences among the 25 highly variable human norovirus genotypes can be expected because the glycan binding site is hypervariable. As the human microbiome varies from person to person, the magnitude and/or specificity of the bacterial help will likely also vary between individuals.

## Mouse Mammary Tumor Virus Uses Bacterial LPS for Immune Evasion

A role for the intestinal microbiota in virus transmission was reported for mouse mammary tumor virus (MMTV), which is vertically transmitted from mothers to their offspring via mother milk. MMTV is a member of the *Retroviridae* family that also includes the human pathogens human immunodeficiency virus (HIV) and human T-cell leukemia virus (HTLV), but there is no human counterpart of MMTV. To establish a new infection, this virus has to pass through mucosal surfaces that are rich in microbiota. It was recently reported that depletion of the commensal microbiota in pregnant mice by broad-spectrum antibiotics has a profound modulating effect on MMTV transmission to the first offspring, even though the mothers continued high level MMTV production in the milk (11). In a complementary approach, wild-type mice transmitted MMTV much more efficiently to their offspring than germ-free mice (11). Furthermore, reconstitution of the germ-free mice with a defined bacterial community restored their ability to transmit MMTV, which may implicate a general bacterial product, e.g., LPS as a general constituent of the outer membrane of gram-negative bacteria, instead of a specific bacterial strain. These combined results support a role of the microbiota in MMTV transmission through the oral route.

The putative role of LPS was studied in more detail. MMTV ultracentrifugation led to comigration of LPS in the gradient. LPS concentration by MMTV likely occurs in specific compartments without affecting the global IL-10 levels, an immunosuppressive cytokine. Furthermore, this interaction potentiated the ability of LPS to induce IL-10 production via engagement of the toll-like receptor 4 (TLR4)-IL-6 cascade. TLR4-dependent IL-10 production was already known to be required for MMTV persistence (12), but it remained unclear whether MMTV proteins or products of the intestinal microbiota triggered TLRs. HIV-1 belongs, like MMTV, to the Retroviridae, and is also transmitted to infants breastfed by HIV-positive mothers. Many biological variables of this transmission route have been reported, e.g., bile-salt stimulated lipase (BSSL) (13), but a role of the commensal microbiota in retroviral pathogenesis should not be dismissed too early. Many studies addressed the impact of the vaginal biome on sexual transmission of HIV-1 (14, 15).

## Poliovirus Needs LPS to Infect Cells

Poliovirus, the causative agent of poliomyelitis, is a human enterovirus and member of the family of the *Picornaviridae*. Poliovirus spreads via the fecal–oral route and will thus encounter a multitude of microbes in the human gastrointestinal tract, before it can establish infection. In fact, orally acquired poliovirus performs a single replication cycle in the gastrointestinal tract before dissemination throughout the body. In 95% of cases, only a transient primary viremia occurs and the poliovirus infection remains asymptomatic. In about 5% of cases, the virus spreads and replicates in other sites such as reticuloendothelial tissue and muscle, causing minor symptoms like fever, headache, and sore throat. Paralytic poliomyelitis occurs in <1% of poliovirus infections and occurs when the virus spreads into the central nervous system.

Early studies indicated that the intestinal microbiome promotes poliovirus replication and pathogenesis. For instance, antibiotic-treated mice were less susceptible to poliovirus disease and demonstrated minimal virus replication in the intestine (16). However, the mechanism remained unclear for some time. Robinson et al. recently demonstrated that bacterial LPS binds to poliovirus particles, thereby increasing the virion stability and infectivity (17). Simple incubation of poliovirus particles with LPS (or peptidoglycan) delayed heat-induced release of the RNA genome and thus maintained a higher infectivity of the virions compared to mock-treated samples. Both molecules enhanced the attachment of radiolabeled poliovirus to cells expressing the poliovirus receptor or to the purified receptor protein.

The authors subsequently identified a specific amino acid residue substitution in the viral capsid that affects virion stability and the sensitivity to LPS-mediated stabilization, possibly by a direct effect on LPS binding (17). The Threonine-to-Lysine substitution at position 99 is one of the attenuating mutations in live-attenuated poliovirus vaccine strains and was reported to affect LPS binding in some experimental settings. The mutant and wild-type viruses behave identical with respect to cell attachment, replication, and pathogenesis in mice, but the mutant virus was unstable in feces, thus causing an environmental fitness cost. Here, the virus uses the microbiota, more specifically a bacterial product (LPS), to enhance its environmental stability, thus promoting transmission to a new host.

## Reovirus also Needs Bacterial Help

The *Reoviridae* is a family of segmented, double-stranded RNA viruses like rotavirus that can affect the gastrointestinal and respiratory tracts. “Reo” is derived from respiratory enteric orphan viruses, which relates to the fact that a disease association was not immediately apparent for some of these viruses. Most cases in adults are mild or subclinical, but rotavirus can cause severe diarrhea and intestinal distress in children.

A link with the microbiota first became apparent in the above referenced poliovirus study in antibiotic-treated mice, which were not only protected against poliovirus, but also against the pathogenic effects of reovirus infection (16). Although reovirus does not induce disease in wild-type mice, overt symptoms are apparent in immunocompromised mice. Reovirus replication causes obstruction of the biliary tract, resulting in hardened feces, but also pathology like enlarged Peyer’s patches. These effects were not observed in antibiotic-treated mice, with a concomitant reduction of the reovirus titers compared to untreated animals. Mechanistic details of the microbiota-induced protection remain currently unknown.

## Common Themes and Remaining Questions

Microbiota are advantageous for the host for several reasons, e.g., for homeostatic functions of the mucosa and for limiting bacterial pathogen colonization. However, recent reports suggest that viral pathogens may utilize the microbiota to boost their replication and/or transmission. Four examples of pathogenic viruses that exploit the surrounding microbial community were discussed. It is likely that commensal bacteria may contribute to the transmission of an array of viruses, not only enteric pathogens, and that this list will grow in the near future. The main players and their functional interplay are summarized in Table 1. Norovirus interacts with glycans on the surface of the intestinal *E. cloacae* to facilitate attachment to the target B cells. MMTV virion particles are stabilized by interaction with LPS, thus boosting the transmission rate. Poliovirus studies indicate that bacterial-primed virus shows enhanced receptor binding. The reovirus studies are not yet very conclusive, but the antibiotic effect on disease induction is apparent. For all cases, some important mechanistic details are still lacking that will be addressed in future research.

**Table 1:** **Pathogenic viruses that exploit intestinal bacteria**.

Virus	Enhancer	Mechanism	Impact
Norovirus	Enteric bacteria interfere with IFN-λ	Facilitate entry block Innate immunity	Boost replication
MMTV	Bacterial LPS	Immune evasion	Boost transmission
Poliovirus	Bacterial LPS	Virus stabilization	Boost transmission
Reovirus	Unknown	Unknown	Promote disease

Although a limited number of virus–bacterial tandems have been studied thus far, it appears that orally transmitted viruses do efficiently exploit the gut microbiota in one way or the other for transmission. Microbiota is predominantly located in the gut and we reviewed virus–bacterial interactions that occur at this location, but bacteria are also found at numerous other sites on the human body, including the skin, respiratory tract, and urogenital tract. Different viruses do navigate one or more of these sites, arguing that functional virus–microbiota interactions may be much more common than previously anticipated. Next generation sequencing techniques are likely to revolutionize the study of this type of cross-kingdom virome–microbiota interactions, e.g., the interplay of the human papillomavirus with cervicovaginal microbiota (18). Although we focused on virus–bacterial synergy, intestinal microbiota can also antagonize viruses and play a protective role, as for instance been reported for rotavirus and influenza virus (19–21). Other pathogens may also exploit the intestinal microbiota for their propagation. For instance, intestinal microbes induce egg hatching of an intestinal nematode in mice (22). Enteric viruses interact with commensal bacteria via recognition of surface glycans and this boosts – directly or indirectly – viral replication.

Microbiota effects may also be indirect via their interaction with the innate and adaptive immune systems (23). Certain components of the microbiota have been shown to trigger inflammatory responses, whereas others induce anti-inflammatory responses. To add to the complexity, viral infections may also affect the microbiome composition. For instance, human studies of airway microbiota demonstrated that the composition of the respiratory microbiota can be modified by viral infection (24).

Understanding how the microbiota interacts with viruses at the molecular mechanistic level may inform future vaccine strategies and the development of novel antiviral drugs. In fact, given this close viral–bacterial collaborations, antibiotic treatment may – on top of the antibacterial effect – impose an indirect antiviral effect. The therapeutic use of bacteriophages may, through their impact on components of the microbiota, indirectly affect their viral counterparts that infect mammalian cells.

## Conflict of Interest Statement

The author declares that the research was conducted in the absence of any commercial or financial relationships that could be construed as a potential conflict of interest.
